# Association of Longitudinal Oral Microbiome Activity and Pediatric Concussion Recovery

**DOI:** 10.3390/microorganisms13020320

**Published:** 2025-02-01

**Authors:** Justin Ceasar, Deepika Pugalenthi Saravanan, Brennen A. Harding, Steven D. Hicks

**Affiliations:** 1 Department of Internal Medicine, Allegheny General Hospital, 320 E North Ave, Pittsburgh, PA 15212, USA; 2 Department of Pediatrics, The Pennsylvania State College of Medicine, 700 HMC Cres Rd, Hershey, PA 17033, USA

**Keywords:** traumatic brain injury, mTBI, saliva, oral–gut–brain axis

## Abstract

Mild traumatic brain injury (mTBI) results in a constellation of symptoms commonly referred to as a concussion. It is unclear why certain individuals experience persistent symptoms. Given the growing evidence linking the microbiome with cognition and inflammation, we examined whether longitudinal microbiome patterns were associated with concussion symptoms. A cohort study of 118 children (aged 7–21 years) was conducted. Symptoms were assessed at three timepoints post-injury (4, 11, and 30 days) using the Post-Concussion Symptom Inventory. Saliva microbial activity was measured at each timepoint using RNA sequencing. A linear mixed model assessed the relationship between microbial activity and symptom burden while controlling for age, sex, and days post-mTBI. The participants’ mean age was 16 (±3) years. The symptom burden decreased across all three timepoints (25 ± 22, 13 ± 17, and 5 ± 12). The longitudinal symptom burden was associated with elevated activity of *Lactobacillus* (F = 5.47; adj. *p* = 0.020) and *Saccharomyces* (F = 6.79; adj. *p* = 0.020) and reduced activity of *Micrococcus* (F = 7.94, adj. *p* = 0.015). These results do not establish a causative relationship, or support the use of microbial measures as a concussion test. Further studies are needed to explore the role of the gut–brain axis in mTBI.

## 1. Introduction

A mild traumatic brain injury (mTBI), or concussion, is the most common form of traumatic brain injury (TBI) [1]. Concussions are typically caused by a hit to the head or body that results in the brain moving quickly back and forth within the confinements of the skull [2]. An mTBI can cause a range of symptoms such as mood and cognitive impairment, lack of coordination, and nausea. While for most individuals, symptoms resolve within 7–10 days post-injury, this may not always be the case, and symptoms may continue to persist. Multiple or repeated mTBIs, especially when occurring in close proximity to one another, tend to have more severe symptoms and a longer recovery period [2,3,4]. However, no clear, succinct protocol exists for predicting symptom severity and the length of the recovery time on an individual basis for mTBIs.

A growing field of research has found a correlation between alterations in the gut microbiome and mTBIs. The human gut microbiome refers to the microorganisms that reside in the digestive-tract and play a vital role in metabolism, nutrition, and immune function. There are approximately one thousand different bacterial species that comprise the gut flora and have a symbiotic relationship with the human body [5]. Changes, or dysbiosis, of the gut microbiome have been associated with many diseases, varying from gastrointestinal pathology to neurogenerative disorders, such as Parkinson’s and Multiple Sclerosis [6]. Perturbations in the oral microbiome are also associated with neuropsychiatric and neurodegenerative disorders [7,8]. The oral–gut–brain axis has been implicated in Alzheimer’s disease, depression, and psychological stress [9,10,11]. These disorders are believed to arise, in part, due to elevated bacterial metabolites and pro-inflammatory markers in the gut that can cross the blood–brain barrier, resulting in central nervous system dysfunction [12]. Based on the understanding of the gut microbiome and its role in human physiology, recent research findings have indicated that gut microbiome dysbiosis may also be strongly linked with mTBI symptom severity and recovery time [13].

Though there are a limited number of studies currently published that focus on the correlation between gut microbiome dysbiosis and mTBIs, there exists a shared consensus that there are changes in the bacterial population in the gut post-TBI [8]. Longitudinal research involving collegiate football athletes over the course of their sports season has assessed microbial changes post-concussion [14]. For the players with a diagnosed concussion, there was depletion of two bacterial species—*Eubacterium rectale* and *Anaerostipes hadrus*—in the gut microbiome compared to those without a concussion. Both species have been shown to have anti-inflammatory properties; thus, their depletion may be associated with peripheral inflammation [14]. In another study that compared the fecal microbiome of moderate/severe TBI patients with healthy control subjects, a decrease in *Prevotella* and *Bacteroides* species and an increase in *Ruminococceae* species was identified in a population of TBI patients. The imbalance between the bacterial species in TBI patients could be explained by altered inflammation and the disruption of gut motility, resulting in the depletion/overgrowth of different bacterial species [15]. 

When considering treatment options based upon the gut microbiome dysbiosis identified by the aforementioned studies, it was found that pre/probiotic supplementation post-TBI improved neurologic function and reduced the time spent in the intensive care unit for patients with a severe TBI [16]. In an additional 2020 study, a correlation between abnormal growth hormone (GH) secretion and gut microbiome dysbiosis in TBI patients was identified. In patients that were treated with recombinant human GH, symptom improvement was noted while patients were on the treatment with recurrence after treatment cessation [17]. However, more research is required to better understand diagnostic and therapeutic implications of microbial dysbiosis for mTBIs.

The current research in TBIs has been heavily focused on recurrent or severe, single-occurrence TBIs. There are limited studies that focus primarily on mTBIs and their association with gut microbiome dysbiosis. Another gap in the literature is the limited number of human studies. While there has been a heavy focus on TBIs induced in mice and rat populations, few studies have investigated if the same correlations are reflected in humans with TBI. In addition, human studies have primarily focused on adults, with limited focus on children and adolescents. Finally, all human studies have focused on the gut/fecal microbiome, with little emphasis on the oral microbiome and its relationship to the gut–brain axis. The oral microbiome may have the potential to provide further insight into alterations in the microbiota and expand our understanding of the various bacterial species affected post-TBI.

In this study, we focus on pediatric patients affected by mTBI to understand changes in longitudinal oral microbiome patterns and their association with concussion symptom persistence and severity one-month post-injury. Considering the identified connection between the neuronal axis and gut biome identified in the above literature, we hypothesized that RNA levels from select oral microbes could be used to monitor and predict recovery in pediatric patients who experience a concussion. This study aimed to address the knowledge gap regarding pediatric populations and longitudinal oral microbiome patterns in mTBI.

## 2. Materials and Methods

This study protocol was reviewed and approved by a centralized Institutional Review Board (Western IRB 1271583) [18]. Written informed consent was obtained from all the participants, and for individuals under 18 years of age, written assent was also provided. This study was registered with ClinicalTrials.gov (accessed on 19 December 2024) (NCT02901821) [19].

This multicenter investigation involved 118 participants aged 7–21 years with a confirmed clinical diagnosis of mTBI, per the 2016 Concussion in Sport Group guidelines [20]. The participants were enrolled from emergency departments, sports medicine facilities, urgent care centers, specialized concussion clinics, and outpatient primary care clinics within 14 days of the injury. The assessments included symptom evaluations, balance testing, cognitive performance, and salivary microbiome profiling. Each participant completed assessments at three timepoints: (1) <2 weeks post-injury; (2) 1 week after enrollment; and (3) ≥3 weeks post-injury. All the measures were conducted within 60 days post-injury. Based on the self-reported symptom scores, the participants were categorized into persistent post-concussive symptoms (PPCSs; *n* = 30) and non-PPCS groups (*n* = 88). The PPCS classification was defined ≥21 days post-injury as a Post-Concussion Symptom Inventory (PCSI) score ≥ 5, which exceeds the 95% confidence interval of age-matched controls without mTBI, as we have previously reported [21]. This categorization resulted in a PPCS prevalence (25.4%) consistent with the prior literature [22,23]. The control participants without mTBI were not included because this study sought to examine the relationship between concussion-related symptoms and oral microbiome activity in the post-injury period.

The participants were enrolled across six institutions: Adena Health System (*n* = 17), Colgate University (*n* = 18), Penn State College of Medicine (*n* = 64), SUNY Buffalo Medical University (*n* = 3), SUNY Upstate Medical University (*n* = 1), and Vanderbilt University (*n* = 15). The exclusion criteria encompassed non-English speakers, individuals with severe neurological injuries (e.g., intracranial bleeding, spinal cord injury, and skull fractures), periodontal disease, upper respiratory infections, secondary oropharyngeal injuries, baseline sensory impairments, substance dependencies, active use of antibiotics, delayed clinical presentation (>14 days post-injury; *n* = 17), incomplete symptom data for PPCS classification (*n* = 106), or falling outside the age range (*n* = 15). The statistical power calculations using the library(pwr) code in R indicated that this study’s sample size (N = 118) provided 99% power to detect an R^2^ value ≥ 0.20 with α set at 0.05 when performing linear regression with up to 4 predictors.

The participant demographic and medical data were collected via surveys, with parental assistance for children ≤12 years old. Symptoms were self-reported using the PCSI [24], enabling the calculation of the symptom burden (total number of symptoms endorsed, out of 22 symptoms) and severity score (total severity of all 22 symptoms, measured on a 6-point Likert scale). Balance and cognitive functions were assessed using the ClearEdge system (Quadrant Biosciences Inc., Syracuse, NY, USA) [25]. Body sway was measured across eight stances with variations of eyes open/closed, feet apart/tandem, and foam pad present/absent. Mean balance performance was calculated for the eight positions. Cognitive tasks included simple reaction time (averaged across two trials), procedural reaction time, and go/no-go testing. Missing values (1852/108,941, 1.7%) were imputed using the k-nearest neighbor method.

The saliva samples (*n* = 354) were collected using OraCollect swabs, and RNA sequencing was performed at a depth of 10 million reads per sample. The sequencing data were first aligned to build hg38 of the human genome using the Bowtie2 aligner and the Refseq database in Partek Flow. The remaining unmatched reads were then aligned to the human microbiome database at the genus level using Kraken. The microbial RNAs were mapped to the Kegg Orthology (KO) database and analyzed in MicrobiomeAnalyst [26]. Quality control parameters were applied, including the total read count, GC content, and average read quality score. Samples with ≥2 parameters outside the acceptable threshold were re-run. The microbial features were filtered for sparsity, yielding 266 genera. Finally, the microbial dataset underwent quantile normalization and mean-center scaling for statistical analysis (Table S1).

A nonparametric repeated-measures analysis of variance (ANOVA) was used to assess changes in symptoms, balance, and neurocognition across the three timepoints for all the participants. The medical/demographic features were compared between the PPCS and non-PPCS groups using *t*-tests, ANOVA, or nonparametric Wilcoxon Rank-Sum testing, as appropriate. The differences in the symptom trajectories between the PPCS and non-PPCS groups were assessed by measuring the interaction effect of the PPCS status and the days since injury on the symptom burden, symptom severity, balance scores, and neurocognitive measures, via linear regression. The differences in transcriptional activity (RNA levels) for the 266 microbial genera were compared between the PPCS and non-PPCS groups at each timepoint using Wilcoxon Rank-Sum tests with multiple testing corrections via the false detection rate (FDR) method. The relationships between microbial activity and continuous symptom measures (i.e., symptom burden, symptom severity, balance score, and neurocognitive scores) were assessed with Spearman rank correlations with FDR correction. Finally, a linear mixed model fit by restricted maximum likelihood (REML) was used to assess the relationship between microbial activity and total symptom burden over time. The participant ID served as the clustering variable. The effect of the interaction between each microbial genera and days post-injury on symptom severity was assessed while controlling for age and sex as covariates. Microbial genera were selected for inclusion in the final model using a feed-forward technique, where only microbes that displayed a significant association with symptom trajectory (*p* < 0.05) were retained. A similar process was then performed using KO abundance, in order to assess the relationship between microbial metabolism and symptom trajectories. All the statistical tests were performed in Jamovi (V2.6) or Metaboanalyst (V6.0) [27].

## 3. Results

### 3.1. Participants

The participants had an average age of 16.4 (±3.4) years (Table 1). The majority were male (69/118, 58.5%) and non-Hispanic (35/38, 92.1%). Approximately half were White (55/118, 57.6%), and one-third (42/118, 35.5%) had suffered a prior concussion. Few (17/118, 14.4%) reported dietary restrictions. Enrollment occurred, on average, 4.5 (±3.4) days post-injury, and there was no difference in enrollment timing across sites (*p* < 0.05). Follow-ups occurred, on average, 11.7 (±4.1) days and 30.1 (±8.5) days post-injury, respectively. The most common mechanism of injury was sport-related concussion (90/118, 76.2%). Few participants reported loss of consciousness (20/118, 16.9%) or amnesia (35/118, 29.7%) in the immediate post-injury period.

### 3.2. Concussion Symptoms

**Figure 1:**
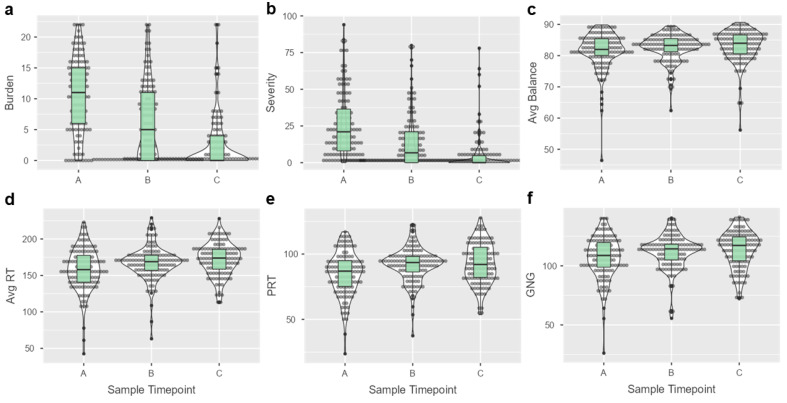
Symptoms, balance, and neurocognition change across follow-up timepoints. The violin and box plots display changes in (**a**) symptom burden, (**b**) symptom severity, (**c**) average (avg) balance score, (**d**) avg reaction time (RT), (**e**) procedural reaction time (PRT), and (**f**) go/no-go (GNG) performance across three timepoints for 118 individuals with concussion. All the measures displayed significant changes (*p* < 0.05) from enrollment (A, 4.5 days post-injury) to the first (B, 11.7 days post-injury) and second (C, 30.1 days post-injury) follow-up visits on nonparametric repeated-measures ANOVA.

The most common symptoms at enrollment were headache (98/118, 83%) and pressure in head (82/118, 69.4%). The most common symptoms at the second visit were still headache (66/118, 55.9%) and pressure in head (57/118, 48.3%). At the third visit, the most common symptoms were headache (29/118, 24.5%) and difficulty concentrating (26/118, 22.0%). There was a descrease in both symptom burden (X^2^ = 137; *p* < 0.001), and symptom severity (X^2^ = 146; *p* < 0.001), and an increase in mean balance score (X^2^ = 8.5; *p* = 0.014), simple reaction time (X^2^ = 24.9; *p* < 0.001), procedural reaction time (X^2^ = 32.2; *p* < 0.001), and go/no-go performance (X^2^ = 23.2; *p* < 0.001) across the three timepoints (Figure 1a–f).

### 3.3. PPCS Versus Non-PPCS Participants

**Figure 2:**
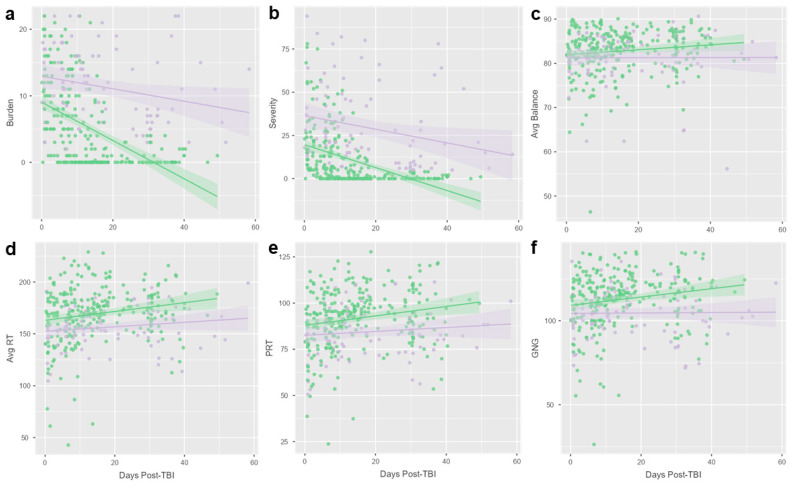
Symptoms, balance, and neurocognition differ between PPCS and non-PPCS groups across follow-up timepoints. Scatter plots and trend lines display changes over time (days post-traumatic brain injury; TBI) for 30 participants with PPCSs (purple) and 88 participants without PPCSs (green). Interactions between PPCS group and time post-injury displayed a significant effect on (**a**) symptom burden, (**b**) symptom severity, (**c**) average (avg) balance score, (**d**) avg reaction time (RT), (**e**) procedural reaction time (PRT), and (**f**) go/no-go (GNG) performance on linear regression analysis.

There were 30 participants (30/118, 25.4%) who met the criteria for PPCSs (PCSI score ≥ 5 three or more weeks after injury). The participants with PPCSs were younger (14.8 ± 3.5 years) than those without PPCSs (16.9 ± 3.2; *p* = 0.004). The participants with PPCSs were more likely to report post-injury amnesia (15/30, 50.0%) than those without PPCSs (20/88, 22.7%; *p* = 0.017). There was no difference in sex, ethnicity, race, rates of prior concussion, or loss of conciousness immediately following injury. The rates of PPCSs were higher among the participants recruited from Penn State (22/64, 34%; *p* = 0.001) but did not differ among the other sites. The interaction between PPCS status and days post-injury had a significant effect on symptom burden (R^2^ = 0.265, F = 63.3, and *p* < 0.001; Figure 2a), symptom severity (R^2^ = 0.265, F = 63.3, and *p* < 0.001; Figure 2b), mean balance score (R^2^ = 0.265, F = 63.3, and *p* < 0.001; Figure 2c), mean simple reaction time (R^2^ = 0.265, F = 63.3, and *p* < 0.001; Figure 1d), procedural reaction time (R^2^ = 0.265, F = 63.3, and *p* < 0.001; Figure 2e), and go/no-go task performance (R^2^ = 0.265, F = 63.3, and *p* < 0.001; Figure 2f).

### 3.4. Microbial Activity—Associations with PPCSs, Balance, and Neurocognition

**Figure 3:**
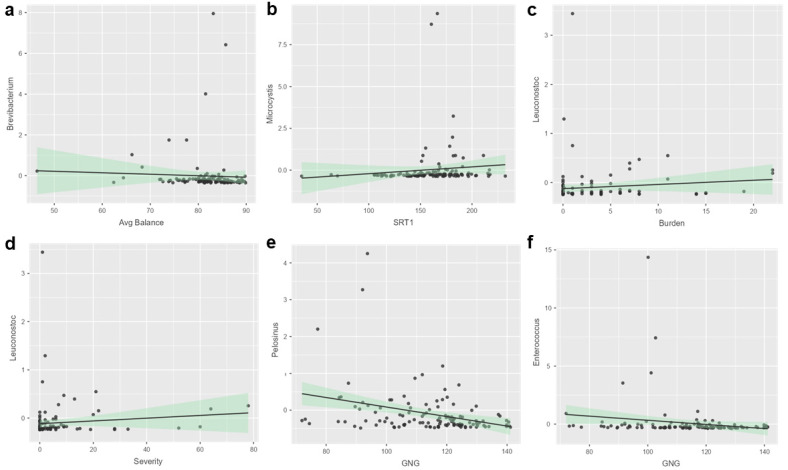
Specific oral microbes display relationships with functional measures of balance and neurocognition. Scatter plots and trend lines display RNA levels of microbial genera associated with performance on balance or neurocognitive testing (N = 118). At enrollment (4.5 days post-injury), there was a significant association of (**a**) average (Avg) balance with *Brevibacterium* and (**b**) simple reaction time (SRT) with *Microcystis*. At the second follow-up visit (30.1 days post-injury), there was a significant association of (**c**) symptom burden and (**d**) symptom severity with *Leuconostoc*, as well as go/no-go (GNG) performance with (**e**) *Pelosinus* and (**f**) *Enterococcus*. All the results include both PPCS and non-PPCS participants and use Spearman rank correlations with false detection rate correction.

There were no microbial genera with significantly different (adj. *p* < 0.05) activity between the PPCS and non-PPCS groups at enrollment or the two follow-up visits (Table S2). A total of 8 microbial genera displayed nominal differences at enrollment, 11 displayed nominal differences at the first follow-up, and 11 displayed nominal differences at the second follow-up, but none survived multiple testing correction. Among all the participants at enrollment (4.5 days post-injury), balance was associated with Brevibacterium activity (Spearman’s R = −0.32 and adj. *p* = 0.043; Figure 3a), and simple reaction time was associated with Microcystis activity (Spearman’s R = 0.29 and adj. *p* = 0.044; Figure 3b). Symptom burden, symptom severity, procedural reaction time, and go/no-go performance did not display a relationship with microbial activity at enrollment. Among all the participants at the second follow-up (30.1 days post-injury), symptom burden was associated with Luconostoc activity (Spearman’s R = 0.31 and adj. *p* = 0.026; Figure 3c), and symptom severity was associated with Luconostoc activity (Spearman’s R = 0.32 and adj. *p* = 0.012; Figure 3d). Go/no-go performance was associated with Pelosinus activity (Spearman’s R = −0.33 and adj. *p* = 0.010; Figure 3e) and Enterococcus activity (Spearman’s R = −0.31 and adj. *p* = 0.020; Figure 3f). Balance, simple reaction time, and procedural reaction time did not display a relationship with microbial activity at the second follow-up.

### 3.5. Microbial Activity and Longitudinal Symptom Trajectories

**Figure 4:**
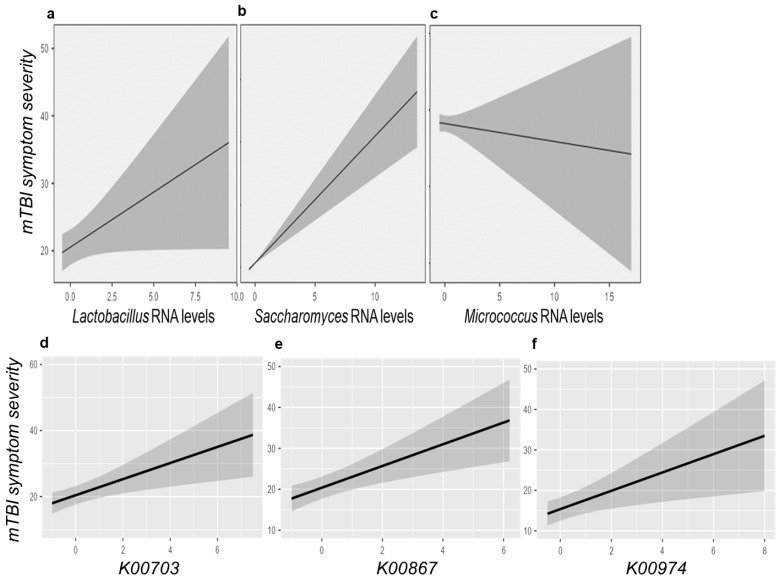
Microbial features associated with concussion symptom trajectories. The marginal mean plots display the levels of (**a**) *Lactobacillus*, (**b**) *Saccaromyces*, and (**c**) *Micrococcus* relative to the concussion severity score on the Post-Concussion Symptom Inventory (PCSI) for 118 individuals with concussion. A linear mixed model demonstrated that activity of these three microbes was significantly (*p* < 0.05) associated with symptom severity over time while controlling for age, sex, and days since injury. A second model examining the relationship of Kegg Orthology (KO) elements identified a relationship between symptom severity and (**d**) K00703, (**e**) K00867, and (**f**) K00974.

A model controlling for participant age and sex (AIC = 2892 and conditional R^2^ = 0.437) demonstrated a significant longitudinal relationship between symptom burden and activity of *Lactobacillus* (F = 5.47 and adj. *p* = 0.020), *Saccharomyces* (F = 6.79 and adj. *p* = 0.020), and *Micrococcus* (F = 7.94 and adj. *p* = 0.015) (Figure 4a–c). Increased levels of *Lactobacillus* and *Saccharomyces* were associated with higher symptoms, whereas elevated levels of *Micrococcus* were associated with reduced symptoms. An additional model employing KO elements (AIC = 2821 and conditional R^2^ = 0.642) demonstrated a relationship between longitudinal symptoms and three KO elements. K00867 (coaA, type I pantothenate kinase, F = 8.21, and *p* = 0.015), K00703 (glgA, starch synthase, F = 4.48, and *p* = 0.035), and K00974 (cca, tRNA nucleotidyltransferase, F = 7.11, and adj. *p* = 0.016) were all associated with higher symptom burden, after controlling for age, sex, and days since injury (Figure 4d,e).

## 4. Discussion

This study identified three oral microbes (*Brevibacterium, Pelosinus,* and *Enterococcus*) that displayed potential associations with measures of neurocognition and balance in the post-injury period. This study also demonstrated the relationship of three oral microbes (*Lactobacillus*, *Saccharomyces*, and *Micrococcus*) with longitudinal symptoms in the 30 days following concussion. The microbial metabolites that displayed associations with symptom severity, after controlling for age, sex, and days since injury, were K00867 (coaA, type I pantothenate kinase), K00703 (glgA, starch synthase), and K00974 (cca, tRNA nucleotidyltransferase). There were nominal differences in the activity of several microbes between the PPCS and non-PPCS groups; however, none of these survived multiple testing corrections. Together, these results suggest that perturbations in the activity of specific oral microbes may reflect evolving concussion symptoms in the post-injury period. However, this study does not provide evidence that microbial activity drives symptomology or predicts future symptom trends.

This study is unique for its inclusion of pediatric participants with mild head injury and its focus on the oral microbiome. In addition, this study’s high-throughput RNA sequencing approach provides a measure of microbial activity rather than microbial abundance. These features differentiate the results from the existing literature examining microbial patterns in TBI [13,14,15,16,17] and likely explain the lack of overlap between our findings and prior studies. Age is known to have a large impact on microbial profiles and may explain some of the differences between our findings and those from prior studies of adults [28]. Intubation, which frequently occurs in severe TBI, can also affect the microbiome [29]. This may explain differences between studies of patients with severe TBI and our study. Finally, though there are similarities between the oral microbiome and the gut microbiome [30], key differences make it unlikely that TBI-related changes would be reflected across both compartments.

There is, however, biologic plausibility between several of the microbial genera identified in this study and inflammatory-related effects on neurobiology. For example, a 2017 study that focused on *Lactobacillus* activity in regard to the body’s inflammatory response found evidence that multiple strains of this microbe were positively associated with an increase in the production of reactive oxidative species and thus an increase in general innate inflammation within the body [31]. This study showed that *Lactobacillus* levels were positively associated with elevated symptom burden over time, suggesting a similar pro-inflammatory effect in the post-concussion period. The previous literature has also displayed initial evidence of *Micrococcus* species being linked to antimicrobial, antioxidant, and anti-inflammatory activity within the human body [32]. This is consistent with findings in the current study, which found higher activity of *Micrococcus* in those with lower symptom burden over time. Further research is needed to confirm these findings and investigate the potential mechanisms by which these microbes exert neurobiologic effects.

The results of this study do not have immediate implications for clinical care in patients with mTBI. Given the variability across various sequencing platforms and alignment strategies [33], the current results should be validated in a separate cohort before firm conclusions can be drawn. Even then, interventional studies are needed to determine whether a true cause-and-effect relationship exists between microbial activity and post-concussion symptoms. However, the current study does provide initial evidence for the feasibility of salivary microbiome measures in pediatric patients with mTBI. The results also suggest that oral microbial activity may be linked to longitudinal concussion symptom burden, representing a novel target for future investigations of management and therapeutic strategies.

The strengths of this study include a well-phenotyped cohort, including granular measures of both subjective and objective symptoms over time. For example, balance, go/no-go testing, and cognitive testing provided objective symptoms that displayed associations with oral microbe activity. Repeated measures of symptoms and microbes over time strengthened this study’s power and provided opportunities to assess the microbiome as a marker of recovery rather than a simple diagnostic. In general, this study’s population of PPCS and non-PPCS participants are reflective of the pediatric population with mTBI, with the exception that rates of LOC and prior concussion were notably similar between the two groups.

Several other limitations exist. First, when examining different microbial organisms, our research looked at genera of organism, not other classification levels. By looking at further classification levels, future data may be able to provide supporting evidence of a more specific microbial species that can be correlated with concussive symptoms. Individuals without mTBI were not included in this study. The inclusion of such a “control group” in future studies could help determine whether the microbes identified in this investigation are specific to mTBI or more generally related to concussion-like symptoms (i.e., sleep difficulties, attention difficulties, and headache). Additionally, since mTBI is an acute, unplanned event, it is impossible to orchestrate fasting sample collections at enrollment. Unfortunately, this dataset did not include granular data about participant dietary habits, so it is unclear if this factor impacted the results. We note that the rates of PPCSs were higher at one of the six recruitment sites—likely because participants at this site were more likely to experience complex mTBI symptoms and require concussion specialty care. Therefore, minor differences in site-specific collection protocols may have influenced the results. Future studies could investigate how a multitude of concussion-related factors (e.g., prior concussion, LOC, and amnesia) and individual characteristics (e.g., BMI, diet, and sleep) impact symptom trajectories or differ among concussion phenotypes.

## 5. Conclusions

By longitudinally assessing symptom severity and oral microbiome changes in children ages 7–21 with a diagnosis of mTBI, this study identified specific oral microbes whose activity was associated with symptom trajectory in the post-injury period. Symptom burden displayed a relationship with *Lactobacillus*, *Saccharomyces*, and *Micrococcus* activity over time. We caution that these results do not establish a causative relationship and do not support the use of microbial measures as a diagnostic or prognostic test for concussion. Further studies are needed to explore the role of the gut–brain axis in mTBI symptom persistence and severity.

## Figures and Tables

**Table 1:** Participant medical and demographic characteristics.

Characteristic	All (N = 118)	PPCS (*n* = 30)	Non-PPCS (*n* = 88)
Age, yrs mean (SD)	16.4 (3.4)	14.8 (3.5)	16.9 (3.2)
Sex, male, *n* (%)	69 (58.5)	14 (46.7)	55 (62.5)
Ethnicity, non-Hispanic, *n* (%)	35 (92.1) ^1^	5 (83.3)	30 (93.7)
Race, White, *n* (%)	55 (57.6)	13 (43.3)	42 (47.7)
Dietary restriction, *n* (%)	17 (14.4)	5 (16.6)	12 (13.6)
BMI, kg/m^2^ mean (SD)	24.2 (5.8)	22.4 (5.9)	24.8 (5.7)
Prior concussion, *n* (%)	42 (35.5)	12 (40.0)	30 (34.1)
Sport-related concussion, *n* (%)	90 (76.2)	13 (43.3)	77 (87.5)
LOC, *n* (%)	20 (16.9)	8 (26.7)	12 (13.6)
Amnesia, *n* (%)	35 (29.7)	15 (50.0)	20 (22.7)

^1^ Note: Ethnicity was available for only 38 participants. Abbreviations: Body mass index (BMI); loss of consciousness (LOC).

## Data Availability

The original contributions presented in this study are included in this article/Supplementary Material. Further inquiries can be directed to the corresponding author(s).

## Supplementary Materials

The following supporting information can be downloaded at https://www.mdpi.com/article/10.3390/microorganisms13020320/s1: Table S1: Microbial concussion dataset; Table S2: Differences in 266 oral microbes between PPCS and non-PPCS participants.
